# Temporal Change of Extracellular Matrix during Vein Arterialization Remodeling in Rats

**DOI:** 10.3390/jcdd6010007

**Published:** 2019-02-02

**Authors:** Ayumi Aurea Miyakawa, Vinícius Bassaneze, Nubia Esteban Duarte, Thais Girão-Silva, Monica Nunes Bizerra, Julliana Carvalho Campos, Jose Eduardo Krieger

**Affiliations:** Laboratory of Genetics and Molecular Cardiology/LIM 13, Heart Institute (InCor), University of São Paulo Medical School, São Paulo 05403-000, Brazil; vinicius@bassaneze.com (V.B.); nued03@gmail.com (N.E.D.); thaisgirao@yahoo.com.br (T.G.-S.); nunesmonica@yahoo.com.br (M.N.B.); jullianacc@hotmail.com (J.C.C.)

**Keywords:** vein arterialization, microarray, extracellular matrix, collagens

## Abstract

The global expression profile of the arterialized rat jugular vein was established to identify candidate genes and cellular pathways underlying the remodeling process. The arterialized jugular vein was analyzed on days 3 and 28 post-surgery and compared with the normal jugular vein and carotid artery. A gene array platform detected 9846 genes in all samples. A heatmap analysis uncovered patterns of gene expression showing that the arterialized vein underwent a partial transition from vein to artery from day 3 to 28 post-surgery. The same pattern was verified for 1845 key differentially expressed genes by performing a pairwise comparison of the jugular vein with the other groups. Interestingly, hierarchical clustering of 60 genes with altered expression on day 3 and day 28 displayed an expression pattern similar to that of the carotid artery. Enrichment analysis results and the network relationship among genes modulated during vein arterialization showed that collagen might play a role in the early remodeling process. Indeed, the total collagen content was increased, with the augmented expression of collagen I, collagen IV, and collagen V in arterialized veins. Additionally, there was an increase in the expression of versican and Thy-1 and a decrease in the expression of biglycan and β1-integrin. Overall, we provide evidence that vein arterialization remodeling is accompanied by consistent patterns of gene expression and that collagen may be an essential element underlying extracellular matrix changes that support the increased vascular wall stress of the new hemodynamic environment.

## 1. Introduction

Coronary artery bypass graft surgery is a widely used procedure to revascularize the ischemic myocardium. The saphenous vein graft is a commonly used conduit, but its effectiveness is limited by acute thrombotic events or late neointimal formation and accelerated atherosclerosis. The patency of a saphenous vein graft is approximately 50% 10 years after surgery, and the overall functionality lasts for 8–10 years [1].

The morphological changes that occur during vein graft arterialization have been well described; however, the molecular mechanism of the whole process has not been fully investigated. We and others have demonstrated the decreased cellular density associated with apoptosis as an early response in vein arterialization remodeling [2,3]. Recently, the observed increase in cell proliferation has been ascribed to the adaptation to the new hemodynamic environment [4,5]. The uncontrolled proliferative process may contribute to graft occlusion or act as a foundation for the development of atherosclerotic lesions. In terms of extracellular matrix, it has been described upregulation of TGF-β associated with increased expression of collagen I and collagen III [6]. Also, there is modulation of proteases that degrade extracellular matrix such as MMP-2 and MMP-9 [7]. The extracellular matrix is very dynamic and its remodeling is essential to adapt the vessel to the increased hemodynamic stress in vein graft procedure. A thorough understanding of the molecular mechanisms underlying these morphological changes may lead to new therapeutic approaches that make vein grafts more resistant to thrombosis and atherosclerosis.

In this study, we performed temporal high-throughput screening of the gene expression associated with the observed structural changes that occur during vein arterialization remodeling in the rat. We found that the pattern of gene expression of arterialized jugular veins is still close to vein expression profile although more similar to the expression profile of the carotid artery. We also found that the extracellular matrix is remodeled during the process of arterialization, and collagens may have a central role in orchestrating early molecular events.

## 2. Materials and Methods

### 2.1. Vein Graft Arterialization Model

Venous arterialization was performed via the end-to-end connection of the rat jugular vein and carotid artery, as previously described [2]. Wistar rats (250–350 g) were anesthetized with a mixture of ketamine (50 mg/kg) and xylazine (10 mg/kg). The right external jugular vein was connected to the common carotid artery by end-to-end anastomosis using a 10.0 silk suture (Ethicon Inc., Somerville, NJ, USA). Arterialized vein segments were collected 3 days (*n* = 3) and 28 days (*n* = 3) after surgery. Normal jugular veins (*n* = 5) and carotid arteries (*n* = 2) were used as controls. This arterialization vein model is well established in our laboratory, with morphological characterization up to 90 days after arterialization [2].

All animal procedures followed institutional guidelines for the care and use of laboratory animals. This study protocol was approved by the local ethics committee (SDC–2253/03/047, CAPPesq–418/03).

### 2.2. RNA Isolation and Microarray Gene Expression Profiling Experiment

Total RNA was isolated using Trizol Reagent according to the manufacturer’s instructions (ThermoFisher Scientific, Waltham, MA, USA). Microarray experiments were performed using the CodeLink^TM^ Expression Bioarray System (GE Healthcare Bio-Sciences, Pittsburgh, PA, USA) according to the manufacturer’s instructions (this platform was acquired by Applied Microarrays, Inc., Tempe, AZ, USA). Briefly, the poly(A)+ RNA (mRNA) subpopulation of the total RNA population was primed for reverse transcription by a DNA oligonucleotide containing the T7 RNA polymerase promoter 5′ to a d(T)24 sequence. After second-strand cDNA synthesis, the cDNA served as the template for an in vitro transcription (IVT) reaction to produce the target cRNA. IVT was performed in the presence of biotinylated nucleotides to label the target cRNA. This method produces approximately 1000- to 5000-fold linear amplification of the input mRNA. A set of bacterial mRNA controls is included in each CodeLink iExpress Assay Reagent Kit to serve as an overall platform performance control group and can also be used to estimate the sensitivity of RNA detection.

Microarray data were prepared using the Codelink R package [8] supplied during the R Bioconductor project [9]. CyclicLoess normalization, the most effective method for normalizing CodeLink Bioarray data [10], was used. MAplot showed the adequate adjustment of the whole dataset (Figure S1). The CodeLink system has 33,849 probes on the microarray. However, the study was performed with 9846 genes that satisfied data quality control criteria (filtering for signals with good intensity and removing genes with a low-intensity signal in at least 50% of the samples of each group).

All microarray files have been deposited in NCBI’s Gene Expression Omnibus (GEO) [11] and are accessible through GEO Series accession number GSE103151 (https://www.ncbi.nlm.nih.gov/geo/query/acc.cgi?acc=GSE103151).

### 2.3. Principal Component Analysis (PCA)

Using Principal Component Analysis (PCA), the sources of variation present in the microarray data that summarize features were analyzed, allowing the visualization and confirmation of clustering results. The aim of the analysis is to reduce the dimensionality of a dataset consisting of a large number of interrelated variables while retaining as much intrinsic variation as possible. This is achieved by transformation to a new set of uncorrelated variables—the Principal Components (PCs)—which are then ordered so that the first few retain most of the variation present in all of the original variables [12].

### 2.4. Clustering Analysis

The pvclust package was used to classify genes into groups (clusters) according to their expression similarities [13]. This package uses a bootstrap analysis for assigning measures of accuracy to estimate samples. It calculates probability values (*p*-values) for each cluster using bootstrap resampling techniques; two types of *p*-values are generated: the approximately unbiased (AU) *p*-value and bootstrap probability (BP) value. The BP value of a cluster is the frequency at which it appears in the bootstrap replicates. Multiscale bootstrap resampling was implemented in the package for calculating AU probability values (*p*-values).

The key idea is that performing computations on the data itself estimates the variation in statistics computed from the same data. Also, heatmaps were generated using the R add-on package pheatmat [14] to reflect gene expression values in several conditions. The technique was applied using Manhattan (also called city block) distance and the Average cluster method.

### 2.5. Multiple Comparisons

To determine differentially expressed genes, we used the double filtering procedure with fold change and statistical tests [15]. Fold change (FC) was calculated by dividing the gene expression means by the jugular-related gene expression mean (ratio). If this number was less than one, the negative reciprocal (−1/ratio) was listed. Statistical tests were performed using multiple comparisons of each gene’s expression between different classes. All possible comparisons within and between the classes were carried out to provide detailed information about the differences in means.

For the analysis, we used the multcomp package from the R statistical environment [16], which provides a convenient interface for the general approach adopted here for simultaneous inference. An average fold change higher or less than ±2.5 and a *p*-value ≤ 0.01 was the cutoff for defining statistically relevant differences for further analysis.

### 2.6. Pathway Analysis of Vein Arterialization Remodeling

Significantly differentially expressed genes (*p* ≤ 0.01) were further analyzed for functional relevance by using Ingenuity Pathway Analysis (IPA) software (version 26127183; Qiagen, Redwood City, CA, USA). The significance of a functional pathway/network was determined by the *p*-value calculated using the right-tailed Fisher’s exact test. The total number of molecules in the reference set (knowledgebase) and test dataset (experimental), as well as the total number of functions/pathway-eligible molecules from each category, were considered for the *p*-value calculations. The effect of a significantly differentially expressed transcriptional regulator was predicted by a z-score assigned by IPA, which is able to infer the activation state (increased or decreased) of a functional pathway/network. The z-score is the normal random variable of a standard normal distribution and indicates how many standard deviations an element is from the mean value. The z-score can be calculated from the following formula: z = (X − μ)/σ, where z is the z-score, X is the value of the element, μ is the population mean, and σ is the standard deviation. If the number of elements in the set is large, about 68% of the elements have a z-score between −1 and 1, about 95% have a z-score between −2 and 2, and about 99% have a z-score between −3 and 3. In our analysis, the z-score is particularly suitable for predicting whether upstream regulatory molecules are activated or inhibited, because the z-score serves as both a significance measure and a predictor of the activation state of the regulator [17].

### 2.7. Real-Time Quantitative PCR Analysis (qPCR)

Total RNA was isolated with Trizol Reagent, and cDNA synthesis was performed with SuperScript III Reverse Transcriptase according to the manufacturer’s instructions (Thermo Fisher Scientific, Waltham, MA, USA).

Collagen expression was evaluated by the TaqMan^®^ Gene Expression Assay (Thermo Fisher Scientific, Waltham, MA, USA). Collagen genes have a very similar DNA sequence. We used predesigned TaqMan assays to specifically detect collagen I (*Col1a1*, Rn01463848_m1), collagen IV (*Col4a1*, Rn01482927_m1), collagen V (*Col5a1*, Rn00593170), collagen VIII (*Col8a1*, Rn01474796_m1), collagen XVI (*Col16a1*, Rn01520480_m1), and collagen XVIII (*Col18a1*, Rn01428995_m1). Only collagen I, collagen IV, and collagen V reached an acceptable efficiency of amplification, so their expressions were evaluated. Collagen VIII, collagen XVI, and collagen XVIII were not detected under the tested conditions.

cDNA was used for the real-time RT-PCR reaction (TaqMan Gene Expression Master Mix, Applied Biosystems, Foster City, CA, USA) in an ABI Prism 7500 Fast Real-Time PCR System (Applied Biosystems, Foster City, CA, USA). All samples were assayed in triplicate. The control Gapdh gene (Rn01775763) was used to normalize the results. The comparative threshold cycle (CT) method was used for PCR data analyses. CT indicates the fractional cycle number at which the amount of amplified target reaches a fixed threshold, and ΔCT is the difference between the threshold cycle for the target (Collagen) and the reference (Gapdh). The levels of collagen gene expression were determined using 2^−ΔΔCT^, where ΔΔCT is the ΔCT value subtracted from ΔCT of the normal jugular vein. Thus, the levels of collagen expression are relative to its expression in the jugular vein.

### 2.8. Picrossirus Red Staining

The vein segments were perfusion fixed in situ via cardiac puncture at 80 mmHg with an initial infusion of saline with 14.8 mM KCl, followed by 4% phosphate-buffered formalin. After 24–48 h, the vein segments were dehydrated in graded ethanol baths, immersed in citrisolv, and embedded in paraplast (Oxford, St Louis, MO, USA). Transverse sections (3 mm-thick) were stained with sirius red (1 mg/mL) and analyzed using ImageJ to quantify the intensity of red.

## 3. Results

### 3.1. Global Gene Expression Pattern of Arterialized Jugular Vein

**Figure 1:**
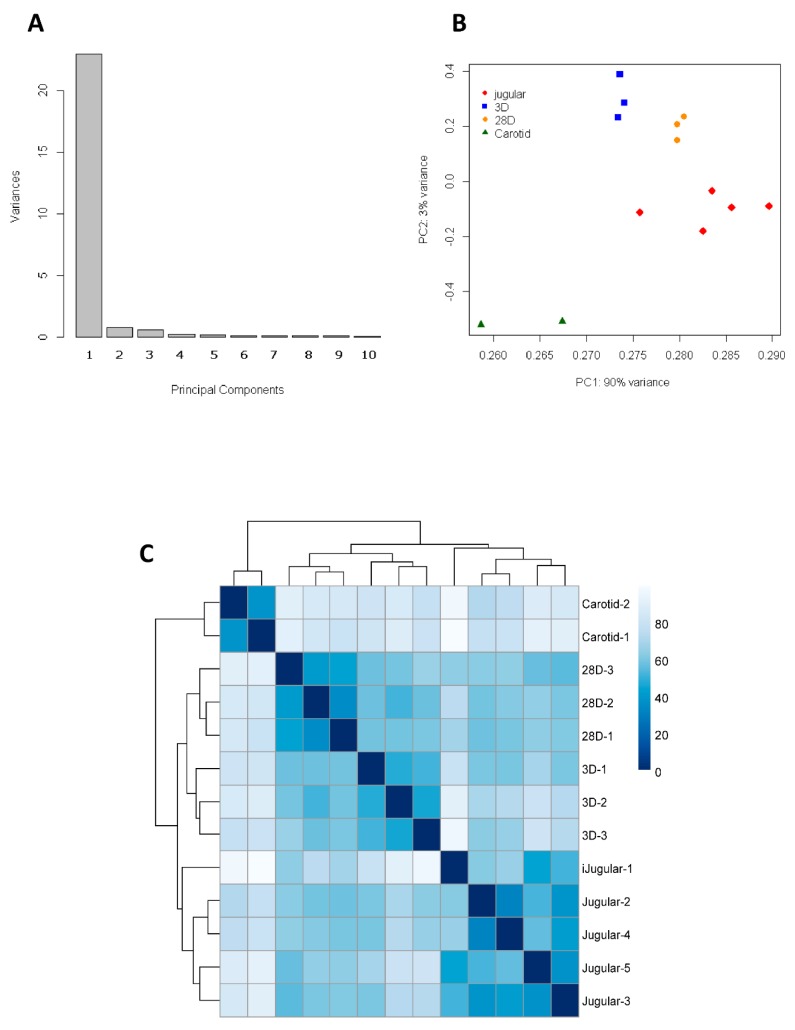
Analysis of 9846 detected genes. (**A**) Plot of eigenvalues of the principal components. Most of the variance in the dataset is contained in the first two principal components (PCs). (**B**) Visualization of the dataset in the subspace of the first two PCs. Jugular vein samples are represented in red, 3D in blue, 28D in orange, and carotid in green. (**C**) Heatmap of sample-to-sample Manhattan distances using the dataset of 9846 genes expressed in all samples of the study. The distances of the experimental source are visualized according to light or dark blue color (low or high correlation, respectively).

To visualize the global gene expression profile during vein arterialization remodeling, we performed microarray analysis in the early (3 days-3D) and late (28 days-28D) stages of the process of arterialization. Normal jugular vein (J) and carotid artery (C) were used as controls. Our first approach was to evaluate the total dataset of 9846 expressed genes by principal component analyses to generate a small number of independent linear combinations (principal components) that capture the variability in the original variables as much as possible; this process simplifies the analysis and visualization of multidimensional datasets. The first two principal components (PC1 and PC2) contained 93% of the variance in the data, allowing most of the information to be visualized in 2 dimensions (Figure 1A), and the PCA plot was able to identify the four experimental groups (J, 3D, 28D, and C), showing the heterogeneity between groups and the homogeneity of the samples within each group. Samples of 3D and 28D was best separated by PC1 while normal jugular vein and carotid artery by PC2 (Figure 1B). Hierarchical clustering analysis showed that the arterialized veins became similar to carotid arteries but were still more closely related to jugular veins in terms of total expressed genes (Figure 1C).

**Figure 2:**
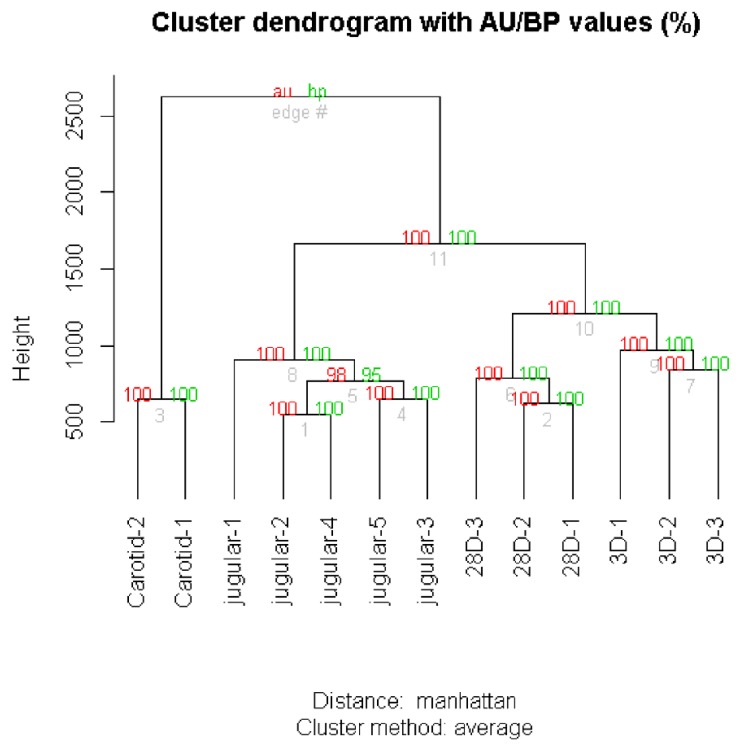
Hierarchical clustering of the differentially expressed genes dataset (1845 genes) determined by multiple comparisons. Values at the branches are AU *p*-values (red), BP values (green), and cluster labels (bottom).

We next selected the differentially expressed gene dataset (1845 genes) identified by multiple pairwise comparisons of the expression level of each gene among the groups considered (J, 3D, 28D, and C). Similar to the observation for total expressed genes (Figure 1C), the dataset of the 1845 identified genes showed that samples in the same experimental group had a similar expression pattern, and arterialized jugular veins had a profile that was more similar to veins than to arteries (Figure 2). To assess the uncertainty in the hierarchical cluster analysis and have greater robustness in the results, we applied multiscale bootstrapping using the pvclust package. With this technique, we verified that the clusters were strongly supported by both the unbiased *p*-value (AU) and bootstrap probability (PB) (Figure 2). In addition, several different methods of clustering analysis produced similar results, as demonstrated by the cophenetic correlation coefficient (Table 1). Overall, it can be argued that the dendrograms appropriately summarize the data, because the correlation between the first distances and the cophenetic distances is high, indicating that the dendrogram preserves the pairwise distances between the original nonmodeled data points.

**Figure 3:**
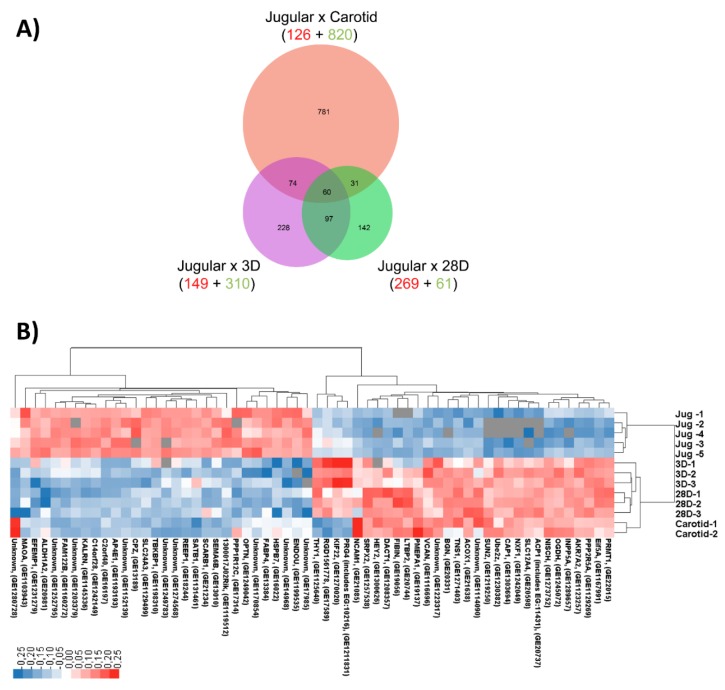
Genes differentially expressed between vein and artery and modulated in arterialized veins foreshadow a directional shift in gene expression for arterialized samples. (**A**) Venn diagram showing the statistically significant differentially expressed genes (1845 genes); (**B**) Hierarchical clustering of the 60 genes differentially expressed among all groups. Downregulated genes are represented in blue, and upregulated genes are represented in red.

Using a Venn diagram, we examined the overlap of differentially expressed genes (Figure 3A). This confirmed that 459 genes were modulated at day 3, and 330 genes were modulated at day 28 compared with normal jugular veins. Additionally, 946 genes were differentially expressed between jugular veins and carotid arteries. Interestingly, hierarchical clustering of the genes modulated during vein arterialization and also differentially expressed between veins and arteries (the 60 genes that intersect the three comparisons studied: J × C, J × 3D, J × 28D) showed that the arterialized jugular vein profile of these genes became similar to the arterial gene expression profile (Figure 3B).

### 3.2. Functional Analysis of Differentially Expressed Genes

**Figure 4:**
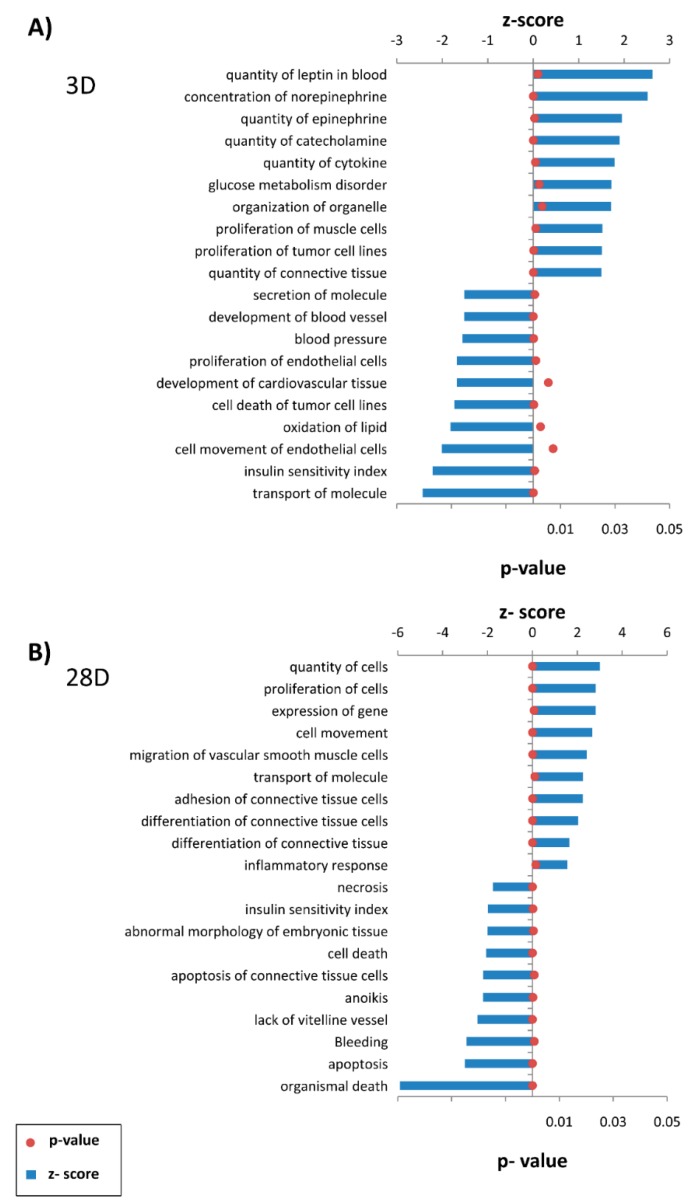
Disease and functional analysis using the IPA knowledgebase for (**A**) 3 days and (**B**) 28 days after the onset of venous arterialization compared with the normal jugular vein. The bars represent z-scores, and the points represent the *p*-value for each disease and function annotated. Diseases and functions with z-scores > 1.5 or z-scores < 1.5 and *p*-value < 0.01 were selected. The complete list is available in Tables S1 and S2.

Genes that were differentially expressed (All-comparison.xlsx file available at Supplementary Material) were analyzed to assess their functional contributions during vein arterialization. We evaluated diseases and functions related to the genes regulated at day 3 (Figure 4A and Table S1) and day 28 (Figure 4B and Table S2). Genes related to the concentration and quantity of catecholamines (epinephrine and norepinephrine) and cytokines were increased at day 3. Genes associated with cell proliferation and processes related to connective tissue were expressed at high level at day 3 and day 28. Additionally, apoptosis was markedly decreased at day 28.

When looking to the 10 most activated and repressed genes, it was observed upregulation of extracellular matrix genes (biglycan, proteoglycan 4 and collagens) at days 3 and 28. The enzyme that participates in collagen assemble (prolyl 4-hydroxylase) is also highly upregulated (Tables S3 and S4).

**Figure 5:**
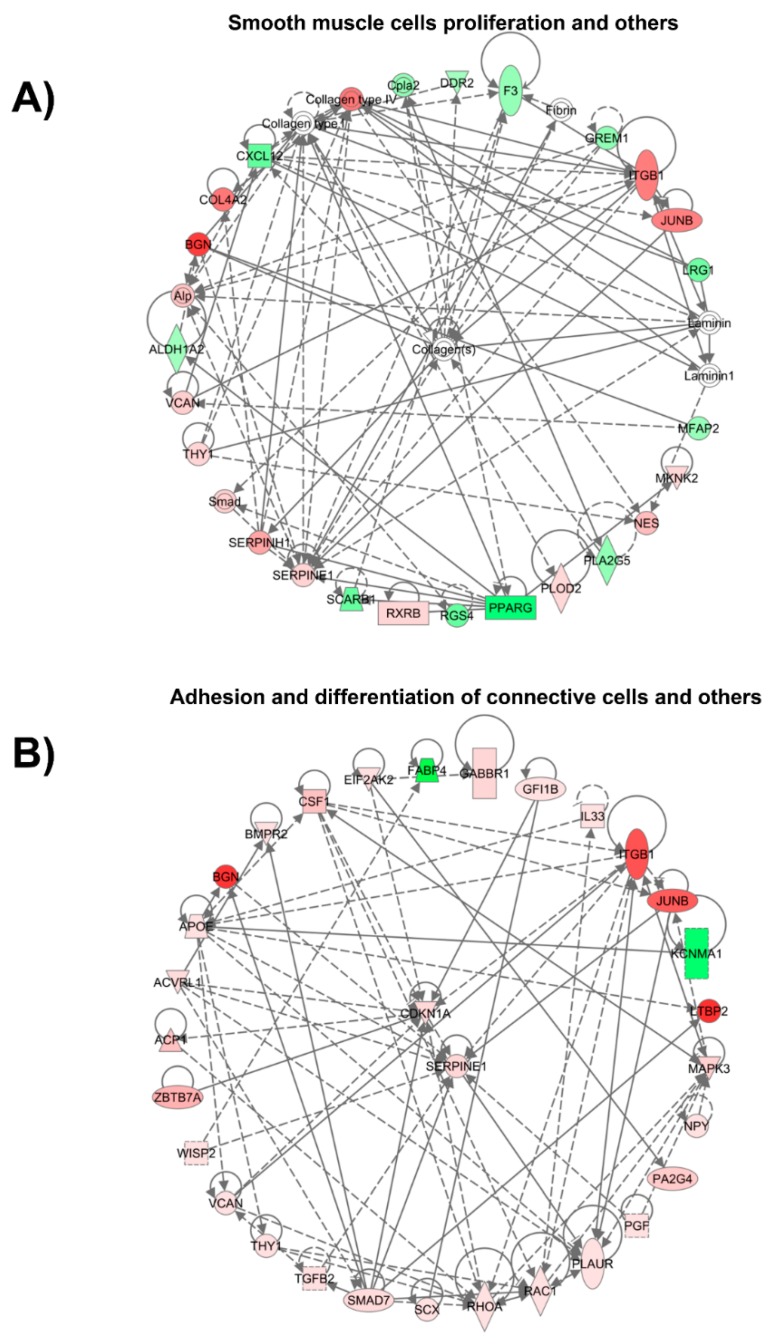
Selected network of genes differentially expressed. (**A**) Network of genes participating in proliferation of smooth muscle cells, cellular assembly and organization, cell function and maintenance, and tissue development at day 3 of vein arterialization remodeling; (**B**) Network of genes participating in adhesion and differentiation of connective cells and differentiation and proliferation of connective tissue at day 3 of vein arterialization remodeling.

To better visualize the relationships among differentially expressed genes, we built a network of differentially expressed genes involved in cellular processes that were regulated at day 3 (cellular assembly and organization, cell function and maintenance, tissue development, and proliferation of smooth muscle cells) and at day 28 (adhesion and differentiation of connective cells and differentiation and proliferation of connective tissue) (Figure 5). Interestingly, in those selected networks, collagen played a key role in cellular function at day 3, and CDKN1A and Serpine 1 had a central role in connective tissue remodeling at day 28.

### 3.3. Extracellular Matrix Modulation during Vein Arterialization Remodeling

**Figure 6:**
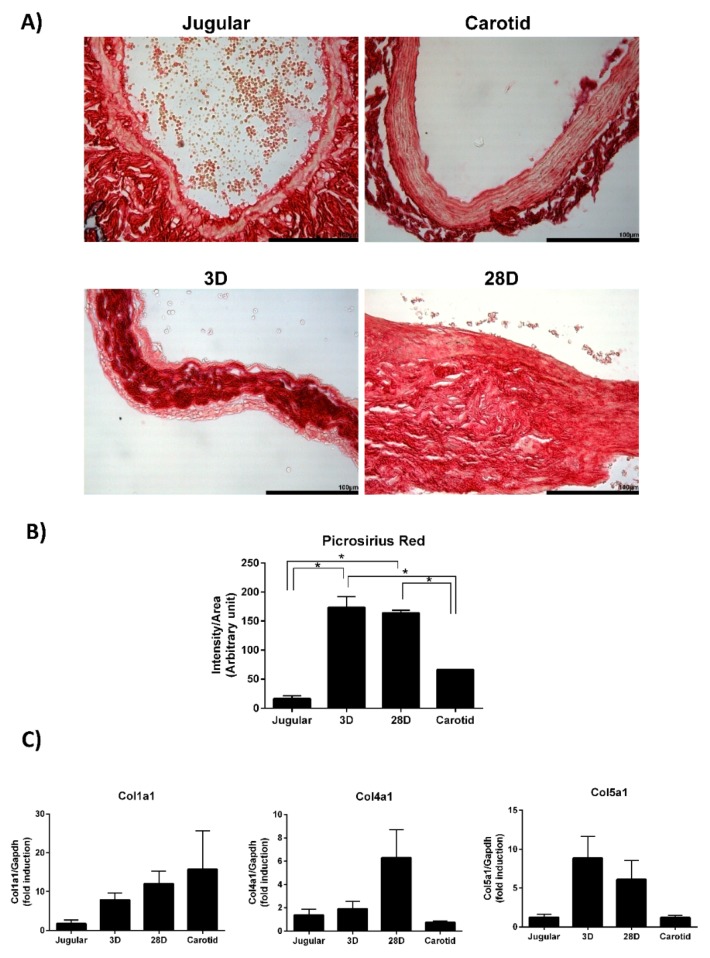
Collagen expression validation. (**A**) Representative images and (**B**) quantification of picrosirius red staining of arterialized jugular vein at 3 days (*n* = 4) and 28 days (*n* = 4). Normal jugular vein (*n* = 3) and carotid artery (*n* = 3) were used as controls. * indicates *p* < 0.05. (**C**) qPCR analysis of collagen genes to validate microarray data. Each bar represents the fold change of collagen expression compared with the level of expression in the jugular vein. Jugular (*n* = 4), 3D (*n* = 14), 28D (*n* = 7), Carotid (*n* = 5). * indicates *p* < 0.05.

The molecular changes occurring during the early stages of vein arterialization impact the whole remodeling process. The network of cellular processes at day 3 (Figure 5A) indicates that collagen makes an important contribution to early molecular changes, so we examined the total collagen content in arterialized jugular veins (Figure 6A). Picrosirius red staining showed a marked increase in collagen at day 3 and day 28 of vein arterialization. We next searched for all types of collagen detected in the microarray experiment. Because different types of collagen are highly similar in their DNA sequence, we validated the collagens that have commercially available TaqMan probes. We observe increased expression of *Col1a1*, *Col4a1*, and *Col5a1*, whereas *Col8a1*, *Col16a1*, and *Col18a1* could not be detected under the conditions tested (Figure 6B).

**Figure 7:**
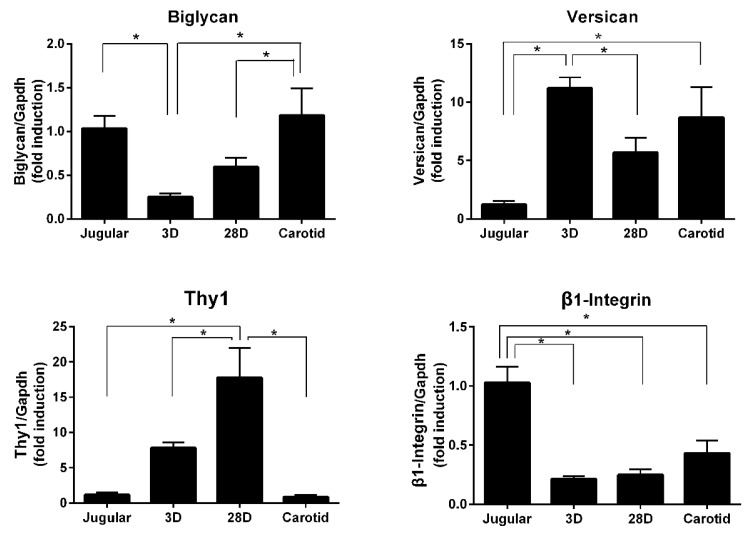
Extracellular matrix-related gene validation. qPCR analysis of versican (Vcan), Thy-1, biglycan, and β1-integrin to validate microarray data. Each bar represents the fold change expression compared with the level of expression in the jugular vein. Jugular (*n* = 4), 3D (*n* = 14), 28D (*n* = 7), Carotid (*n* = 5). * indicates *p* < 0.05.

Of the 60 genes differentially expressed among all groups (J × 3D × 28D × C; Figure 3B), we selected four genes related to extracellular matrix composition and cell–matrix interaction to also be validated. We found increased expression of versican and Thy-1 and decreased expression of biglycan and β1-integrin (Figure 7).

## 4. Discussion

We attempted to analyze the temporal profile of gene expression that is generated during vein arterialization remodeling using a rat model. Our data show that the changing gene expression profile of the arterialized vein becomes similar to that of an artery, and the results highlight the modulation of the extracellular matrix during the adaptive remodeling.

Arteries and veins have functional and anatomical differences; their molecular differences are established before the onset of circulation [18]. During blood vessel development, specific molecular pathways are activated to determine arteries and veins, and distinct profiles of gene expression are established between them. Kudo et al. suggested that a vein placed into arterial circulation loses its venous identity, but it does not gain the arterial identity [19]. This observation was made based solely on the expression of Eph-B4 (venous marker) and Ephrin-B2 (arterial marker). We are now approaching this issue through a broader spectrum. Using high-throughput screening of gene expression, we demonstrate that vein arterialization remodeling results in temporal changes in the gene expression profile; namely, from the expression profile of a vein to that of an artery. Although the global profile of gene expression in arterialized veins is still closer to that of the jugular vein, it is clearly observed that the pattern becomes similar to the pattern of the carotid artery. Interestingly, looking at the genes modulated during vein arterialization and those differentially expressed between veins and arteries (the 60 genes that intersect the three comparisons studied), a pattern of “molecular arterialization” of the jugular vein is observed. Others have performed microarray experiments [20,21,22], but none of included an analysis to evaluate the global change profile during the remodeling process.

Early and late functional events modulated are observed at day 3 and day 28 after arterialization surgery. The quantity of catecholamines is modulated early as an immediate mechanism to control vascular tone. Similarly, cytokines are also increased and have been described as participating in the initiation and progression of the remodeling of arterialized vein [23,24]. Cytokines stimulate immune cell proliferation and differentiation and also induce vascular cell growth and migration. These processes are very well described for vein arterialization remodeling; here, we also detected an increase in cell proliferation and migration both at 3 days and 28 days after surgery. Apoptosis and cell death are markedly reduced at day 28, which is also in accordance with data in the literature [2,25]. Connective tissue modulation is increased during early and late remodeling, with an increase in quantity after 3 days and an increase in adhesion and differentiation after 28 days. In fact, Abeles et al. demonstrated the upregulation of genes involved in the remodeling of the extracellular matrix (tenascin-C, thrombospondin, lysyl oxidases, and osteopontin) when veins are exposed to arterial flow [20]. Interestingly, our data show that collagen is a central player in cellular organization, function, and maintenance. The total collagen content is increased 10-fold in arterialized veins compared with that in normal jugular veins. Our results demonstrate the upregulation of collagen types I, IV, and V gene expression.

Collagen is the most abundant fibrous protein in the interstitial extracellular matrix and provides tensile strength to the vascular wall. Collagen types I and III are the major fibrillar collagen species in the vessel, representing 60% and 30% of vascular collagen, respectively. Collagen III was not detected in the microarray experiment; therefore, we did not explore this gene further. Collagen V is involved in the regulation of fibril assembly and can be classified as a regulatory fibril-forming collagen [26]. It has been reported that collagen V is found in tissues containing collagen I and that the correct collagen fibril formation is affected by the availability of collagen V [27]. We observe increased expression of both collagen type I and V, which indicates the attempt of the vessel to have the correct assembly of collagen fibers to support the increased mechanical stress. These results are in consonance with those obtained using a canine model, which demonstrated increased collagens I, III, and V [22]. Collagen IV is a component of the basement membrane and maintains smooth muscle cells in the quiescent contractile phenotype. We demonstrate that collagen IV expression is upregulated on day 28 but not on day 3 of arterialization. One may speculate that, in the early stages of arterialization, smooth muscle cells are proliferating and migrating to remodel the vessel with the newly synthesized collagens I and V. When mechanical stresses are again equilibrated, collagen IV starts to be produced to return the cells to the quiescent state. It is important to point out that we just recognize differences in collagen gene expression. However, the cellular response depends not only on the concentration and type of collagen to which the cells are exposed but also the conformation and organization of the collagen fibers within the extracellular matrix.

In addition to the fibrillar structure of collagens, proteoglycans are components of the extracellular matrix and provide hydration and compressive resistance. We were able to detect increased expression of versican and decreased expression of biglycan. Versican is associated with cell proliferation and migration and was found to increase in a porcine model of a saphenous vein graft [28]. Biglycan participates in the lateral assembly of collagen fibers and has also been implicated in the inflammatory response as an endogenous ligand of TLR4 (Toll-like receptor 4) [29]. Versican and biglycan are important for organizing the extracellular matrix and may influence cellular adhesion. Our microarray data analyses also reveal the modulation of β1-integrin and Thy-1 expression, which are transmembrane proteins related to cell–cell and cell–matrix interactions. The interplay of those molecules and the influence on vein arterialization remodeling should be further investigated.

The extracellular matrix is highly dynamic and is continuously remodeled to maintain vessel structure and integrity. It interacts with cells and regulates proliferation, migration, and differentiation. The present work shows that, during vein arterialization remodeling, several components of the extracellular matrix are modulated to allow the vessel to compensate for the mechanical stress. A better understanding of how the extracellular matrix regulates the remodeling process, together with information that can be extracted from the high-throughput experiment, will contribute to the development of new therapeutic approaches to increase the patency of vein grafts. Future strategies of intervention need to be done with careful single or combined gene intervention and methodologies to prevent vein graft disease.

## Figures and Tables

**Table 1:** Cophenetic correlation coefficient for different clustering strategies. The closer the values are to 1, the closer they are to the original distances. Different methods of clustering analysis revealed similar results.

Distance Measure	Clustering Method	Cophenetic Correlation Coefficient
City block	Centroid	0.95
	Complete	0.96
	Single	0.97
	Average	0.97
	Ward	0.91
Euclidean	Complete	0.96
	Single	0.97
	Average	0.98
	Ward	0.88

## Supplementary Materials

The following are available online at http://www.mdpi.com/2308-3425/6/1/7/s1, Figure S1. Data quality evaluation. Representative MA plot depicting log-intensity overall expression average data versus log-intensity ratios (A) before and after (B) background correction and cyclic loess normalization. (C) Normalized signal box-plots indicating the lack of significant differences in the log-intensity expression average among every group. Table S1: Disease and function analysis using IPA knowledgebase for 3 days of venous arterialization compared with normal jugular vein. Table S2: Disease and function analysis using IPA knowledgebase for 28 days of venous arterialization compared with normal jugular vein. Table S3: The top 10 genes upregulated and downregulated after 3 days of venous arterialization compared with normal jugular vein. Table S4: The top 10 genes upregulated and downregulated after 28 days of venous arterialization compared with normal jugular vein. All-comaparisons.xlsx: Excel file containing the genes differentially expressed between J × C, J × 3D, J × 28D, C × 3D, C × 28D and 3D and 28D. Each spreadsheet tab has the information of Genebank ID, Codelink ID, fold change, *p*-value and FDR corrected *p*-value for each gene.
