# Supplementary material for: Temporal Change of Extracellular Matrix during Vein Arterialization Remodeling in Rats
Source: J Cardiovasc Dev Dis. 2019 Feb 2;6(1):7. doi: 10.3390/jcdd6010007 (PMC6463024; doi:10.3390/jcdd6010007)
Supplement: Supplementary file 1 [file jcdd-06-00007-s001.zip › Supplementary-edited-R2.docx]

**
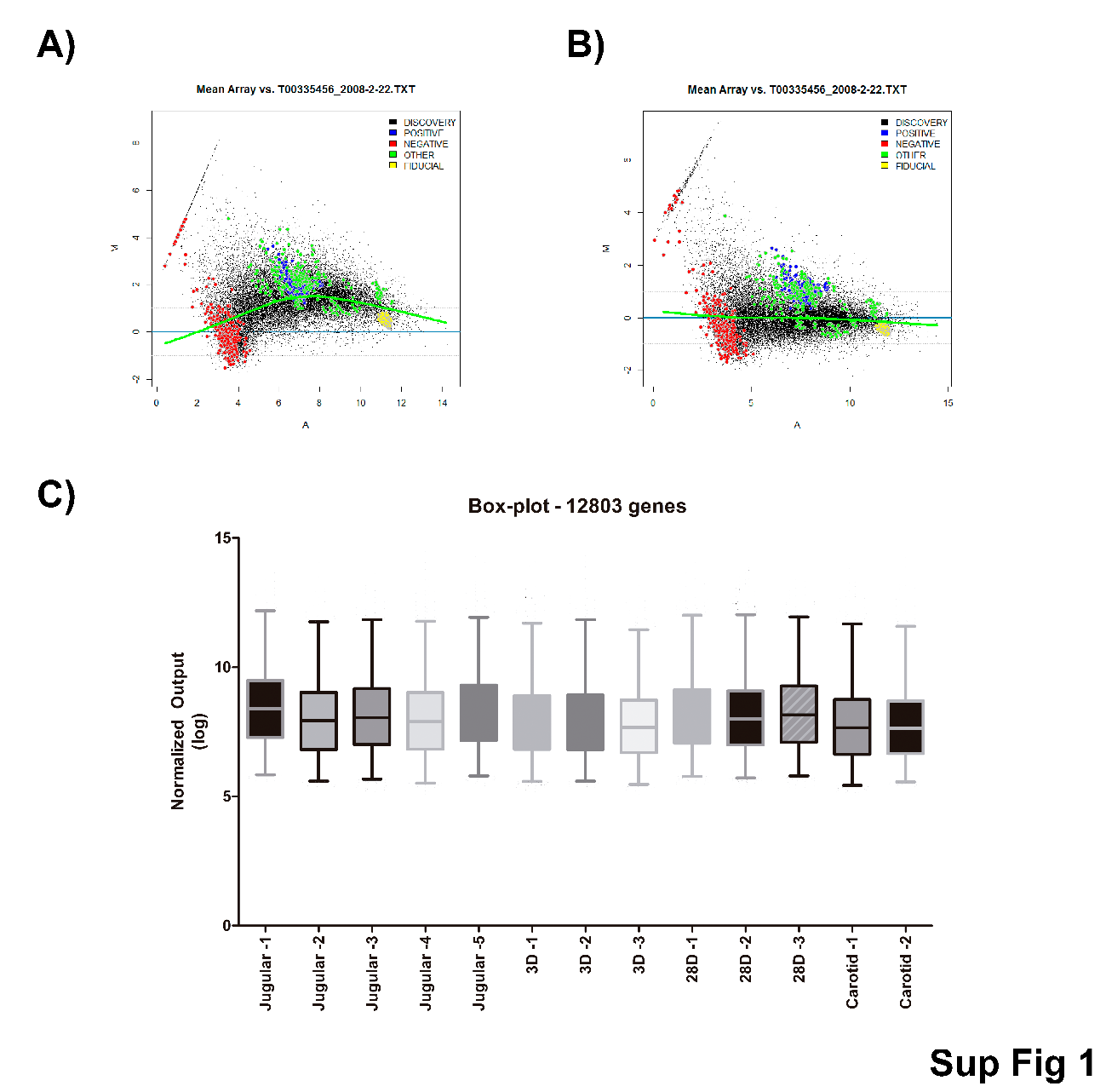
**

**Figure S1.** **Data quality evaluation**. Representative MA plot depicting log-intensity overall expression average data versus log-intensity ratios (**A**) before and after (**B**) background correction and cyclic loess normalization. **(C)** Normalized signal box-plots indicating the lack of significant differences in the log-intensity expression average among every group.

**Table S1: Disease and function analysis using IPA knowledgebase for 3 days of venous arterialization compared with normal jugular vein.**

| DISEASE OR FUNCTION ANNOTATION | p-Value | Activation  z-score | # Molecules |
| --- | --- | --- | --- |
| Quantity of leptin in blood | 1.71E-03 | 2.62 | 7 |
| Concentration of norepinephrine | 8.20E-07 | 2.52 | 9 |
| Quantity of epinephrine | 4.65E-04 | 1.95 | 5 |
| Quantity of catecholamine | 6.13E-06 | 1.90 | 12 |
| Quantity of cytokine | 7.80E-04 | 1.79 | 12 |
| Glucose metabolism disorder | 2.18E-03 | 1.72 | 38 |
| Organization of organelle | 3.27E-03 | 1.72 | 16 |
| Proliferation of muscle cells | 9.21E-04 | 1.52 | 16 |
| Proliferation of tumor cell lines | 1.68E-04 | 1.51 | 48 |
| Quantity of connective tissue | 4.75E-05 | 1.50 | 15 |
| Secretion of molecule | 5.62E-04 | -1.51 | 22 |
| Development of blood vessel | 1.12E-06 | -1.52 | 37 |
| Blood pressure | 1.50E-04 | -1.55 | 16 |
| Proliferation of endothelial cells | 9.53E-04 | -1.68 | 14 |
| Development of cardiovascular tissue | 5.51E-03 | -1.68 | 15 |
| Cell death of tumor cell lines | 2.35E-04 | -1.74 | 49 |
| Oxidation of lipid | 2.67E-03 | -1.82 | 11 |
| Cell movement of endothelial cells | 7.28E-03 | -2.01 | 13 |
| Insulin sensitivity index | 5.14E-04 | -2.21 | 5 |
| Transport of molecule | 4.13E-05 | -2.43 | 52 |

**Table S2: Disease and function analysis using IPA knowledgebase for 28 days of venous arterialization compared with normal jugular vein.**

| DISEASE OR FUNCTION ANNOTATION | p-Value | Activation z-score | # Molecules |
| --- | --- | --- | --- |
| Quantity of cells | 3.52E-06 | 3.01 | 50 |
| Proliferation of cells | 1.95E-13 | 2.82 | 109 |
| Expression of gene | 5.76E-04 | 2.81 | 8 |
| Cell movement | 3.70E-13 | 2.67 | 75 |
| Migration of vascular smooth muscle cells | 7.00E-05 | 2.42 | 8 |
| Transport of molecule | 8.58E-04 | 2.26 | 40 |
| Adhesion of connective tissue cells | 2.75E-05 | 2.25 | 10 |
| Differentiation of connective tissue cells | 1.63E-06 | 2.03 | 25 |
| Differentiation of connective tissue | 5.52E-06 | 1.65 | 26 |
| Inflammatory response | 1.25E-03 | 1.56 | 23 |
| Necrosis | 6.66E-08 | -1.76 | 75 |
| Insulin sensitivity index | 1.94E-04 | -1.98 | 5 |
| Abnormal morphology of embryonic tissue | 3.72E-04 | -2.00 | 19 |
| Cell death | 1.83E-10 | -2.07 | 97 |
| Apoptosis of connective tissue cells | 7.10E-04 | -2.20 | 12 |
| Anoikis | 1.91E-04 | -2.20 | 7 |
| Lack of vitelline vessel | 1.52E-05 | -2.45 | 6 |
| Bleeding | 7.00E-04 | -2.94 | 15 |
| Apoptosis | 4.86E-08 | -3.01 | 76 |
| Organismal death | 1.19E-10 | -5.90 | 76 |

**Table S3: The top 10 genes upregulated and downregulated after 3 days of venous arterialization compared with normal jugular vein.**

| Name | Fold Change | P-VALUE |
| --- | --- | --- |
| Upregulated genes |  |  |
| prolyl 4-hydroxylase, beta polypeptide | 19,73 | 3.91E-03 |
| troponin C type 2 (fast) | 14,60 | 5.95E-03 |
| wingless-type MMTV integration site family, member 9B | 14,37 | 1.96E-05 |
| follistatin-like 1 | 14,22 | 2.41E-03 |
| biglycan | 13,74 | 4.35E-04 |
| actin, alpha 1, skeletal muscle | 12,80 | 2.65E-03 |
| ferritin, light polypeptide | 11,02 | 5.20E-05 |
| enolase 3 (beta, muscle) | 10,24 | 9.18E-03 |
| proteoglycan 4 | 9,78 | 5.24E-06 |
| collagen, type IV, alpha 2 | 9,73 | 9.00E-04 |
|  |  |  |
| downregulated genes |  |  |
| proline dehydrogenase (oxidase) 1 | -5,68 | 1.07E-05 |
| RIKEN cDNA 1300017J02 gene | -5,72 | 2.16E-05 |
| cytokine-like 1 | -5,85 | 5.62E-05 |
| synuclein, gamma (breast cancer-specific protein 1) | -5,87 | 4.73E-03 |
| galectin-related inter-fiber protein | -5,91 | 3.07E-03 |
| sulfotransferase family, cytosolic, 1A, phenol-preferring, member 1 | -6,59 | 5.36E-05 |
| cytidine monophospho-N-acetylneuraminic acid hydroxylase, pseudogene | -7,51 | 2.93E-06 |
| endonuclease, polyU-specific | -7,75 | 1.12E-04 |
| aldehyde dehydrogenase 3 family, member A1 | -7,75 | 2.34E-06 |
| flavin containing monooxygenase 2 (non-functional) | -8,89 | 1.27E-04 |

**Table S4: The top 10 genes upregulated and downregulated after 28 days of venous arterialization compared with normal jugular vein.**

| Name | Fold Change | P-VALUE |
| --- | --- | --- |
| Upregulated genes |  |  |
| latent transforming growth factor beta binding protein 2 | 25,45 | 1.24E-05 |
| prolyl 4-hydroxylase, beta polypeptide | 22,02 | 2.23E-03 |
| biglycan | 21,45 | 1.13E-04 |
| follistatin-like 1 | 19,03 | 4.65E-04 |
| integrin, beta 1 | 18,55 | 2.50E-04 |
| jun B proto-oncogene | 17,44 | 5.47E-07 |
| collagen, type VIII, alpha 1 | 14,41 | 1.54E-05 |
| collagen, type IV, alpha 2 | 14,26 | 2.88E-04 |
| wingless-type MMTV integration site family, member 9B | 13,96 | 7.98E-06 |
| ferritin, light polypeptide | 13,23 | 2.72E-05 |
|  |  |  |
| downregulated genes |  |  |
| RIKEN cDNA 1300017J02 gene | -3,35 | 3.67E-04 |
| chromosome 2 open reading frame 40 | -3,38 | 1.04E-06 |
| G0/G1switch 2 | -3,44 | 1.41E-03 |
| MAS1 oncogene | -3,47 | 3.14E-04 |
| family with sequence similarity 122B | -3,56 | 2.98E-05 |
| heat shock 27kDa protein family, member 7 (cardiovascular) | -3,60 | 1.12E-03 |
| T-cell immunoglobulin and mucin domain containing 4 | -3,64 | 4.00E-04 |
| WNK lysine deficient protein kinase 2 | -3,66 | 6.70E-04 |
| endonuclease, polyU-specific | -3,67 | 4.70E-04 |
| proline dehydrogenase (oxidase) 1 | -3,87 | 2.97E-05 |
